# Design and Control of Monolithic Compliant Gripper Using Shape Memory Alloy Wires

**DOI:** 10.3390/s23042052

**Published:** 2023-02-11

**Authors:** Ganapathy Then Mozhi, Kaliaperumal Dhanalakshmi, Seung-Bok Choi

**Affiliations:** 1 Department of Instrumentation and Control Engineering, National Institute of Technology, Tiruchirappalli 620015, Tamil Nadu, India; 2 Department of Mechanical Engineering, The State University of New York, Korea (SUNY Korea), Incheon 21985, Republic of Korea; 3 Department of Mechanical Engineering, Industrial University of Ho Chi Minh City (IUH), Ho Chi Minh City 70000, Vietnam

**Keywords:** monolithic gripper, compliant mechanism, self-sensing, geometric advantage

## Abstract

This paper presents the design, fabrication and testing of a shape memory alloy (SMA)-actuated monolithic compliant gripping mechanism that enables translational motion of the gripper tips for grasping operation suitable for micromanipulation and microassembly. The design is validated using a finite element analysis (FEA), and a prototype is created for experimental testing. The reported gripping structure is simple and easy to build and design. The gripper is demonstrated to have a displacement amplification gain of 3.7 that allows maximum tip displacement up to 1.2 cm to possess good handling range and geometric advantage which cannot be accomplished by conventional grippers. The position of the gripper tip is predicted from the variation in the electrical resistance of the SMA wire based on the self-sensing phenomena. Self-sensing actuation of the SMA allows the design of a compact and lightweight structure; moreover, it supports the control loop/scheme to use the same SMA element both as an actuator and sensor for position control. The geometrical dimensions of the SMA wire-actuated monolithic compliant gripper is 0.09 m × 0.04 m and can be operated to handle objects with a maximum size of 0.012 m weighing up to 35 g.

## 1. Introduction

New and effective technologies involving smart materials have emerged in response to the intensifying demands for precision in all fields of endeavor. Micrometers and even nanometers have replaced millimeters in size due to the inherent advancements of smart materials. This sparked the creation of a new collaboration, shape memory alloys (SMAs), with compliant mechanisms (soft mechanisms without joints) to form a gripping mechanism. The end-effector, the device at the end of a robotic arm, is intended to interact with the environment and is a vital component of the robotic ecosystem as a result of the increasing automation of handling operations. The end effector of a mechanical arm of robots used to grasp objects is often called the robotic gripper. It is important in industrial and medical industries to capture the targets properly and accurately.

Generally, engineering systems are built from discrete components and are intended to be sturdy and rigid. Robotic grippers often use pneumatic actuators or electric motors as their driving force, which necessitates several extra parts, such as stepper motors with gears, ball screws, etc. Grippers driven using hydraulic power require air pumps with pipelines, hydraulic machines with hydraulic cylinders, etc. [1]. This results in the robotic gripper having a series of complicated systems, which increases size and weight, consumes a lot of energy, and increases cost. A statistical examination on the review of the pneumatically operated, parallel grippers with two jaws revealed that, despite the enormous number of grippers in the market, the majority of them have similar traits like small stroke and limited force [2]. Grasping objects using a gripper can be enabled and organized by actuation, controlled stiffness, and controlled adhesion. End-effectors made from flexible and soft components may frequently grasp or operate a wider variety of objects as compared to rigid grippers. Such grippers are an illustration of morphological computation where mechanical compliance and material softness significantly reduce the control complexity. In order to build lighter, easier, and more versatile grippers, advanced materials and soft components including silicone elastomers, shape memory materials, active polymers, and gels are increasingly being investigated [3].

SMAs are frequently used as the driving element in gripping operations to overcome the shortcomings of traditional actuators because of their advantages, such as high power-to-weight ratio and repetitive cyclic actuation. Since the performance of the robotic arm depends heavily on its actuator and configurations, various arrangements of SMA-actuated soft fingers are examined. The SMA wire-operated gripper, in which the claws can be moved angularly to grasp micro-components as small as 500 µm due to the linear displacement provided by the SMA wire, was developed. However, during the deactivation phase, the gripper jaws are released by an additional pair of bias springs attached behind the jaws [4]. Flexure hinges activated by the SMA wires have been used in the gripper prototype, which was reported in [5] to improve the gripper’s functionality; this involves a complex mechanism. The stroke of the gripper is extended by these hinges, which lessens joint stress. To handle micro-components, a unique gripper powered by SMA has also been developed and investigated in [6], in which a single SMA spring is used to open and close the gripper’s claws, which reduces manufacturing costs and facilitates production. The maximum deformation of the spring is 1 cm and hence the movement of the gripping arm is limited to 1 cm; further, the incorporation of fan to cool down the SMA spring and additional sensors at the control loop add cumbersomeness to the system. A gripper with an SMA element for easy load transfer and smooth actuation by incorporating a slider crank mechanism was designed in [7], possessing a limited linear displacement up to 7.23 mm. A novel construction of an SMA micro-manipulator with a gripping mechanism activated by numerous SMA micro-actuators allows a maximum movement of 8.9 mm and suggests that the created SMA micro-manipulator can be employed for applications that demand high-precision coordination and the manipulation of minute entities in constrained spaces like biomedical fields and beyond [8]. The multidirectional bending movement in biomedical applications is achieved using a catheter with multiple SMA actuators [9]. Due to the linear actuation capabilities, the SMA wire is frequently utilized as the tendon-drive actuator in soft finger designs to generate certain bending angles and a tip force of 0.89 N, as mentioned in [10], where each module consists of lengthy SMA wire tendons enveloped using bearings. The holding arrangement of the finger made from polyvinyl chloride (PVC) is softened due to the high temperature SMA in [11]. The gripper being realized using multiple curved SMA actuators in [12] demonstrates that the curved gripper has a lifting force of 1.5 N for 25 mm, which is three times greater than the straight gripper; this concept requires customized SMA actuators. The miniaturized gripping mechanism is realized based on the hand grasping; the distance is controlled by the antagonist configuration of SMA spring and rigid spring while the improved force of 1.10 N is obtained using a finger palm design compared to other designs [13]. Despite the adequate stiffness that this sort of gripper offers, they may produce too much contact pressure, which, in the absence of a sophisticated gripping jaw, could harm clutched objects. 

The transmission mechanism functions as a passive transferring unit by converting the features of the actuator into the desired movement. The transmission mechanism can be a rigid-body which does not store internal energy, is not compliant, and does not have an elastic structure that stores energy. The development and implementation of an experimental test rig is demonstrated in [14], in which a differential mechanism is used with robotic underactuated soft grippers to move multiple robotic fingers independently of one another using just a single actuator, the servomotor. A method for creating 3D-printed, tendon-actuated fingers for a kind of robotic gripper is presented in [15], where each finger is a monolithic device made up of rigid units linked by flexible wave-shaped joints. The methodology facilitates to identify the properties of the parts including thickness, length, wave amplitude, and phase. This feature enables the tendon-driven actuation using the motor, to be used to produce more intricate finger movements in addition to finger flexion. The compliant mechanisms do not need to be assembled from separate parts and can be created in a variety of length scales; the gripper proposed in this article prefers to use these mechanisms that are built as monolithic structures for the design of a gripping module.

The structural designs are robust but flexible and developed as a single integrated system. The microgripper fabricated using thin film SMA provides a maximum gripper opening of 0.5 mm exerting 50 mN force for transporting microelectronics devices safely [16]. Grippers are created with structural compliance to advance towards the adaptive handling of unfamiliar items in unstructured situations as stated by [17,18]. Compliant, soft grippers are useful for handling delicate things of varied sizes because they conform to object shapes through their elastic deformation [19]. They may be made nearly monolithic with the right design, requiring little assembly. A recent class of mechanisms called “compliant mechanisms” use the compliance of the individual parts of the mechanism to convey motion and/or force. Any desired input–output, force-displacement parameters with stiffness limitations can be incorporated into their design. These mechanisms can easily be integrated with smart actuation schemes, such as electrostatic, piezoelectric, and shape memory alloy (SMA) actuators, because of their flexural behavior [20]. 

The proposed design describes a technique for creating robust and flexible jointless compliant mechanisms actuated by the SMA wire to develop an SMA-based gripping system. The SMA wire, used as the actuation device, is located far from the gripper’s tip, yet the effect is conveyed by elastic deformations to the gripping arm, allowing the arms to open and close. Output displacement is several orders of magnitude greater than the stroke of the actuator (displacement amplification) that minimizes the overall size of the gripper. This ensures greater geometrical advantage and hence good mechanical efficiency at the expense of mechanical advantage (gripping force). To retain the structural rigidity of the gripping module, deformation needs to be kept to a limit for a gripping structure. Another requirement for these grippers, in addition to handling various ranges, is the ability to detect deflection during manipulation. Any external sensor would greatly increase the size and complexity of the gripper. A two-finger gripper with differential SMA springs is created and controlled using the Proportional–Integral controller with a two phase control method, however, the scheme requires additional sensors to detect displacement of the actuator [21]. Integrated sensing methods to retain the overall dimension have been implemented in [22], which achieves limited jaw motion up to 32 µm. As mentioned by [23], self-sensing approaches were also developed for piezoelectric, SMA actuators and ionic polymers, to remove the complexity brought on by additional sensors. In the SMA-actuated flexible needle developed in [24], the resistance feedback control of the SMAs facilitate the active needle to reach target points. The self-sensing technique has been employed to deduce the gripper’s displacement feature from its electrical resistance.

Grippers are often large due to the bulky actuation mechanisms; this contribution introduces a compact gripper design using a flexible actuation method by employing the SMA wire. The highlight is that the compliant gripping arrangement offers a displacement amplification, which cannot be accomplished using the conventional design of grippers. Certain grippers developed using the compliant mechanism have limited distance between the gripping jaws and hence handle objects with limited size. The gripper design with dimensions 0.05 m × 0.03 m illustrated in [25] can grip objects with dimensions less than 1 mm. In the proposed design, the force applied at the input end (drive link) causes a displacement which gets amplified at the output end (gripping arm); thereby in comparison with the similar dimension of a conventional gripper, this compliant gripper can hold larger objects. It is worthy to note that the compliant structure integrated with the SMA wire actuator forms a self-biased gripping assembly that exterminates the process for the provision of extra biasing unit. The monolithic architecture, as opposed to other grippers that use multiple parts, simplifies the design and production. Such mechanisms may be employed in the production of hand-held tools, bioinspired devices, MEMS, etc. A small, cost-effective gripping structure is provided by the compliant architecture in combination with the apt actuation mechanism, the SMA wire actuator. The self-sensing phenomena of the SMA wire decreases the complexity of the entire unit, furthermore creating a sensorless monolithic gripper.

## 2. Structural Realization of the Gripping Mechanism

**Figure 1:**
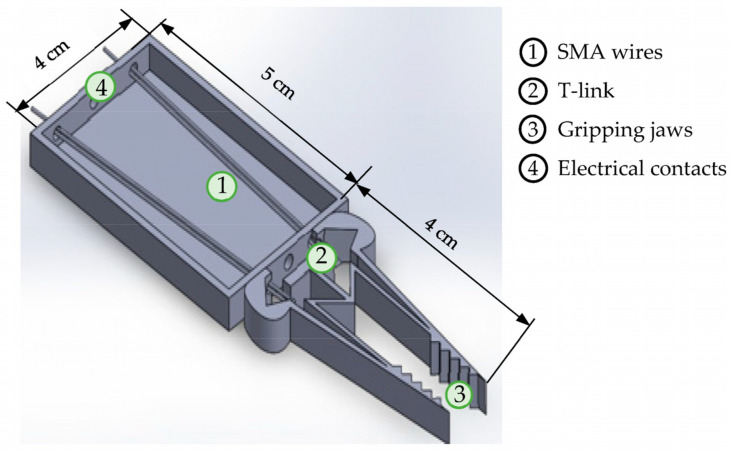
Structure of the gripper: CAD model.

The SMA wire linear actuator, the compliant mechanism, and the gripping claws form the three main structural elements for the assembly of the gripper. To provide a uniform distribution of force to the two jaws of the gripper, the SMA wires are connected at the T-link of the compliant mechanism. The actuation force is transferred via the elastic links to the tip of the gripper and the structure of the gripper design is illustrated in Figure 1. A translational actuator that exhibits a linearly increasing force for a given excitation is required for the operation of the gripper. SMA wires that are attached to both sides of the T-link of the compliant mechanism serve this purpose. Shape memory effect (SME), the prime feature, represents the ability of the SMA to restore the original shape upon joule heating. This effect, also termed as thermally-induced phase transformation, undergoes a reverse-phase transformation (from a detwinned martensite to a austenite state) that completes the shape recovery. Cooling down to a temperature in the absence of stress leads to the formation of the twinned martensite with no associated shape change. However, when the material is cooled with a mechanical stress more than the detwinning start stress, the phase transformation results in the formation of a detwinned martensite, leading to a shape change (stroke). This construction uses a self-biased design to provide adequate force to actively displace the jaws of the gripper with no additional mechanical stress. The SME can be applied in a variety of engineering domains. Upon joule heating, the stroke of the SMA wire actuator is increased due to the high force input exhibited by the SMA [26]. 

In SMA technology, the output force typically increases with increasing force, decreasing the length of the actuator. These actuators can generate relatively consistent responses when used in controlled circumstances; the repeatability is high, standard deviation is too low (virtually invisible) [27]. To be able to handle a wide range of objects, a transmission mechanism is attached downstream of the actuator. The transmission mechanism behaves as a passive transferring module that transforms the SMA wire actuator characteristics into the desirable movement. Rigid-body mechanisms, which store no internal energy, or compliant mechanisms which have an elastic structure that does store energy, can be used to create the transmission mechanisms. Compliant mechanisms formed as a monolithic structure are preferred for the design of the gripper in this contribution. These are constructed with elastic links that store energy so that the applied force is in direct relation to the range of displacement. These mechanisms can be controlled actively by the appropriate control techniques. The topology of the compliant mechanism consists of elastic links that are frictionless joints to undergo elastic deformation for the operation of the gripper. The components of the mechanism are considered beam elements for the calculation of the required force for its operation.

## 3. Analysis of the SMA Wire Integrated Compliant Mechanism

### 3.1. Mathematical Analysis of the Compliant Structure

**Figure 2:**
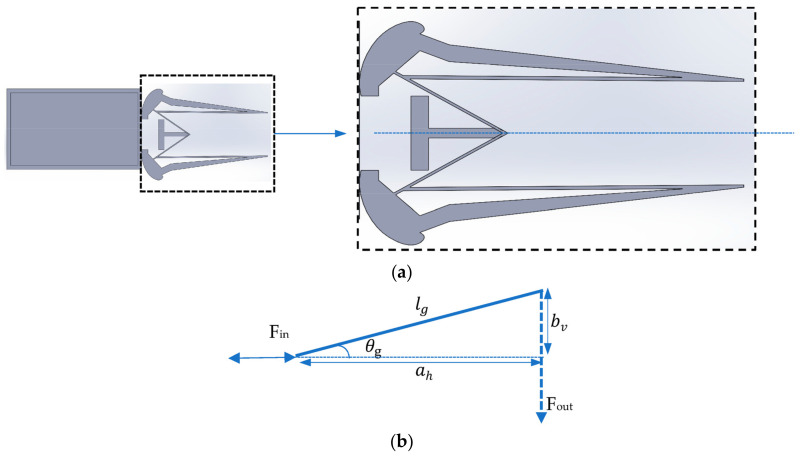
Gripping system: (**a**) symmetrical complaint gripping system, (**b**) upper section of the symmetrical angular gripper.

Motion for performing gripping action is achieved using two SMA wire actuators connected parallel to each other. These are coupled with the transforming mechanism, the compliant structure in series by which the linear motion of the actuator is changed into the intended displacement. Compliant mechanisms use the deformation of elastic elements to generate motion. They offer various benefits, such as the absence of wear and tear or friction and can typically be manufactured as a single part, eliminating the need for assembly, and they also offer improved accuracy. The elastic links responsible for motion can be described using the beam theory represented via the Pseudo-Rigid-Body (PRB) models, as illustrated in [25,28]. The compliant mechanism is made up of elastic links with a drive link that is symmetrically connected to two gripping arms, as shown in Figure 2a. Figure 2b depicts the upper section of the symmetrical angular gripper; the drive link drives/actuates the gripping arms based on the input force (*F_in_*). The compliant assembly is assumed to contain a bar of length $\;l_{g}$, width $b_{g},\;\mathrm{and}\;\mathrm{thickness}\;t_{g}$, with the angle the bar creates being denoted by *θ_g_* and in its initial angular position by *θ_o_* (15°).

The input and output ends act as elastic springs that move with the input force; the horizontal and vertical distances are represented by, $a_{h}$, $\;b_{v}\;$, respectively, and is given using Equation (1).

(1) $$a_{h}=l_{g}cos\theta_{g},\;\;\;\;\;\;\;\;\;b_{v}=l_{g}sin\theta_{g}$$

Approximating the change in input displacement (*U_in_*) by the first-order Taylor series, the following equations can be obtained:

(2) $$\;\;\;\;\;\;\;\Delta a_{h}=\Delta U_{in}=\frac{\partial a_{h}}{\partial \theta }\;d\theta$$

(3) $$\Delta a_{h}=\Delta U_{in}=\left(-l_{g}sin\theta \right)\;d\theta$$

In the above, $\Delta \theta_{g}$ is the small change in the angle. However, the sign denotes the direction of the application of force. Similarly, the approximation of the change in output displacement (*U_out_*) is undertaken by the first-order Taylor series to form Equations (4) and (5).

(4) $$\;\Delta b_{v}=\Delta U_{out}=\frac{\partial b_{v}}{\partial \theta }\;d\theta$$

(5) $$\;\Delta b_{v}=\Delta U_{out}=\left(l_{g}\cos\theta \right)\;d\theta$$

From the above equations, it is evident that the initial angle *θ*_0_ of the gripper plays a vital role in the enhancement of displacement at the output end. To have a displacement gain of 3.7, this angle is chosen as 15°.

Displacement gain $\left(\frac{U_{out}}{U_{in}}\right)$ is a fixed design constraint that is fixed as 3.7 to hold objects of a size up to 0.012 m, bearing a load carrying capacity of 35 g. From Equations (2)–(5), it is evident that the initial angle *θ*_0_ plays a vital role in the enhancement of displacement at the output end. To have a displacement gain of 3.7, this angle is chosen as 15°. The length of the gripper arm is selected based on the maximum deflection required at the jaw. For the desired displacement of 1 cm at the output jaw of the gripper, the input displacement required is around 0.32 cm to achieve the amplification gain of 3.7 from Equations (2)–(5). Angular change is computed as 3° for the maximum input displacement, and so the necessary jaw length of the gripper is estimated to be 4 cm from Equations (3) and (5).

The cantilever beam serves as a useful analogy for elastic spring-like linkages and a simple set of equations govern the behaviour of a straight cantilever beam with a rectangular cross section. The basic ideas behind this kind of beam analysis can be applied to elastic arms of any size and shape under specific assumptions such as small deflections and no yielding. To evaluate the stiffness performance of the flexible gripper arm, it is assumed as a soft manipulator and modelled as a cantilever beam; for a specific degree of deflection, the manipulators provide force and the term “spring rate” or “stiffness of the beam” refers to the ratio between force and deflection. For a deflection at the end of the beam perpendicular to the beam axis, the force can be expressed as in Equation (6).

(6) $$F_{out}\;=\left[\frac{3E_{g}I_{g}}{l_{g}{}^{3}}\right]U_{out}$$

Stiffness of the gripping arm is given using Equation (7).

(7) $$K_{g}=F_{out}/U_{out}=3E_{g}I_{g}/l_{g}{}^{3}$$

Here, $E_{g}\;$ is the Young’s modulus of the gripper arm, $I_{g}$ is the area moment of inertia of the gripper arm, and $l_{g}$ is the length of the gripper arm. It is clear that the stiffness depends on the geometry of the gripper arm as well as the material properties.The simple linear relationships between forces and displacements is applicable under the elastic limit of the material.The material chosen to fabricate the compliant mechanism is Acrylonitrile Butadiene Styrene (ABS), the links are constructed to provide displacement amplification, and its material properties are listed in Table 1.

**Figure 3:**
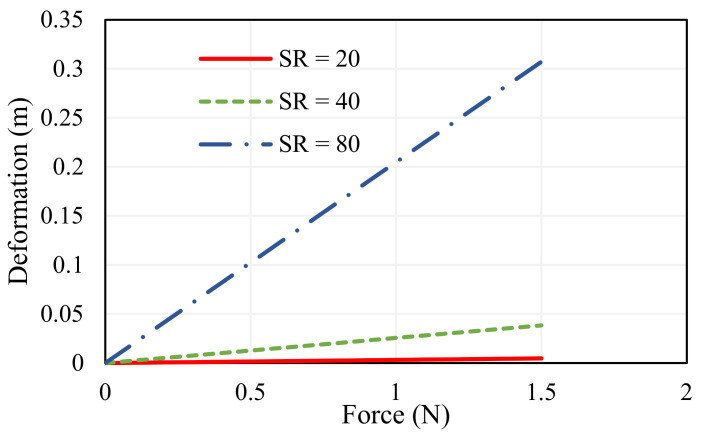
Deflection of the gripping arm for varying slenderness ratio.

The thickness of the compliant mechanism is selected based on the deformation exhibited by the gripper for various slenderness ratios (SR) of the jaws which is given using

(8) $$\mathrm{SR}=l_{g}/t_{g}$$

where $l_{g}$ is the length of the gripper arm (m) and $t_{g}\;$ is the thickness of the gripper arm (m). The thickness of 1 mm offers nominal displacement, whereas beam elements with higher SR offer flexible operation and less SR exhibit sturdiness, or stiff movement, which is portrayed in Figure 3. Experimentation is performed to determine the deformation for varying force and the Root Mean Square Deviations (RMSD) for SR = 20, SR = 40, SR = 80 are determined as 0.023, 0.02, 0.025, respectively. The width of the jaw ($b_{g}$), is selected to be 0.5 cm, which is a significant parameter for grasping the object.

### 3.2. Analysis on the Selection of SMA Wire Actuator

SMA, a class of intelligent material that responds to joule heating, is utilized in a simple wire geometry because of its high-force exertion behaviour. The SMA is attached to the compliant mechanism that transforms the motion and provides a different output displacement as required, reducing the overall complexity of the system. Two SMA wires are connected in parallel to each other on both sides of the T link; each SMA wire is configured as a loop to offer more strain. The strain value essential to provide the necessary displacement determines the length of the SMA wire. The input stroke needed is 0.32 cm to provide the motion required for the mechanism with an amplification gain of 3.7. An SMA length of 10 cm is needed to provide this stroke at the linearization temperature. SMA wires are attached as loops in both of the symmetrical parts of the gripper to shorten the actuation section. As a result, the length of the actuation section is limited to 5 cm. 

The elastic strain energy stored in the links is transferred to perform the work at the output and the strain energy depends on the input parameters as expressed in

(9) $$SE=\frac{1}{2}\;K_{in}U_{in}{}^{2}$$

(10) $$SE=\frac{1}{2}\;F_{in}U_{in}$$

The work produced by the grasping mechanism is equal to the stored strain energy, in accordance with the rule of conservation of energy as stated in Equation (11).

(11) $$\frac{1}{2}\;KU_{in}{}^{2}=F_{out}U_{out}$$

**Figure 4:**
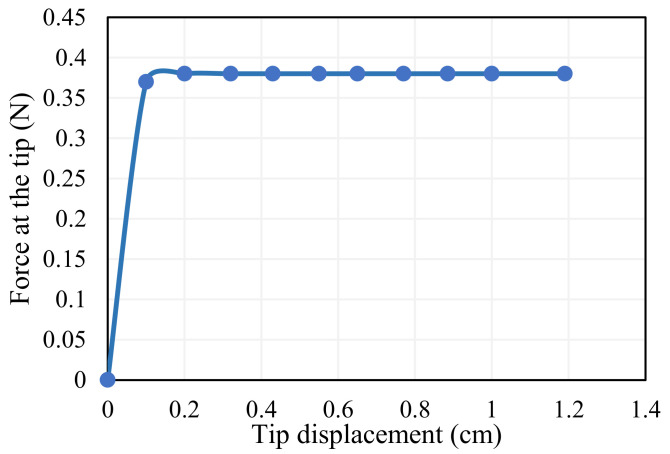
Strain energy due to input work.

The stated relation can be used to calculate the input force needed to operate the gripper for the gripper’s maximum displacement. The input force required is 7.4 times more than the gripper’s output force. The output force of the gripper (*F_out_*) for the displacement of the gripper is observed as 0.38 N from Figure 4. Thus, the maximum input force required to operate the gripper for maximum displacement is 2.8 N. The Dynalloy Inc. SMA wire that complies with this force is 0.15 mm in diameter; this dimension is chosen for the creation of the gripping mechanism.

### 3.3. Analysis Using ANSYS

The finite element method (FEM), an effective numerical tool, is essential for testing the design of any module. Before conducting an experiment, the design is verified and the performance is studied using numerical modelling and simulations. This analysis helps to comprehend the behavior of the mechanism under a specific loading cycle and indicates the possibility for weight reduction and optimization for an overdesigned component. The static structural analysis is performed using the finite element method, where the entity is split up into several manageable chunks called finite elements, and the challenges are resolved by utilizing these chunks. Steps undergone for the analysis of the designed gripper are creating the model, importing the model, definition of the material properties, meshing, applying boundary conditions, and analysis.

**Figure 5:**
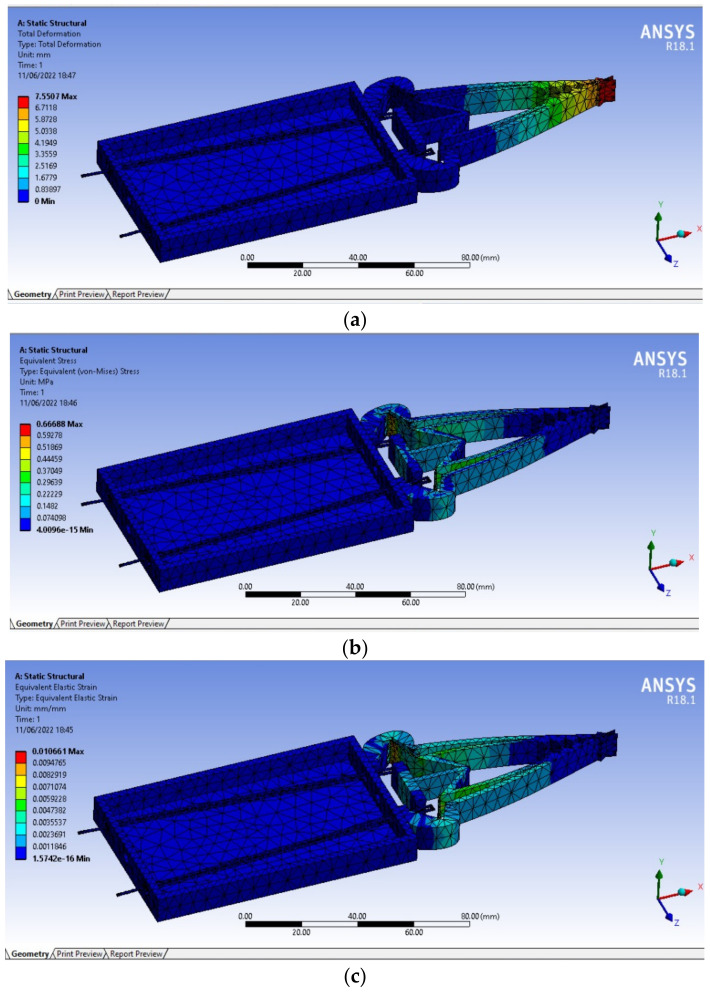
Numerical simulation of the gripping assembly using ANSYS; (**a**) deformation, (**b**) stress, (**c**) strain.

To numerically investigate the behaviour of the gripper, a series of simulations are executed using ANSYS^®^ on a three-dimensional gripper with the different values of input force to the gripper. The simulation is performed to investigate the displacement distribution of the gripper as a function of an input force. From the simulation results shown in Figure 5a, it could be observed that an input force of 2.8 N is required to generate a maximum displacement of 1 cm. Hence the input force is set to 2.8 N by selecting appropriate SMA wire dimensions. It should be noted that this structural simulation of the gripping mechanism gives the displacement profile along the gripper body, and the values indicate the displacement occurring across the gripper. Using Ansys^®^ simulations, it is seen in Figure 5a that there is a negligible rise in displacement near the actuation section, whereas there is a huge rise near the tips of the gripper when actuated with the desirable force; the stress and strain distribution of the structure is depicted in Figure 5b,c. This conclusion further strengthens the analytical formulations for deciding the input force needed for the gripper. The material properties used for the simulation are listed in Table 1.

## 4. Characteristics of SMA Wire and Compliant Mechanism

### 4.1. Characterization of SMA Wire

The SMA wire actuator bearing the dimensions 0.2 mm thickness with a length of 10 cm and the compliant mechanism with the geometrical dimensions specified in Table 1 are analyzed for its characteristics with varying currents. Their characteristics are examined for the identification of functional parameters like the excitation current and frequency of operation. The SMA wire is chosen for the design of the gripping mechanism due to its advantage of higher force compared to the SMA spring. To determine the sensing current and the proper configuration of the grasping assembly, it is essential to identify the fundamental characteristics of the SMA wire. The SMA wire actuator shrinks and enters an austenitic state during joule heating; however, during the cooling cycle it needs more biasing force to return to its deformed martensitic condition. The assembly to which SMA is coupled determines the biasing force; this contribution does not need a separate biasing unit because the compliant mechanism provides the necessary bias to the SMA wire during its martensitic phase. The SMA, on appropriate excitation using the activation current, generates a force that causes displacement on the mechanism to which it is attached. 

**Figure 6:**
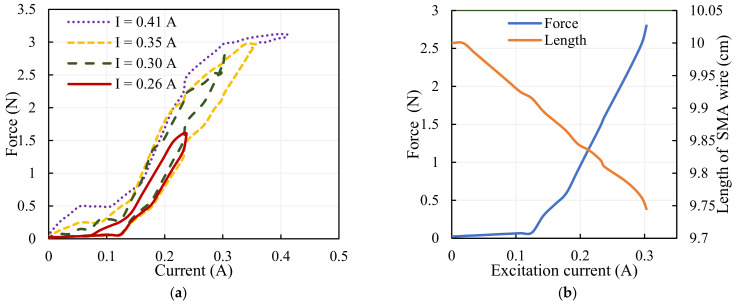
SMA characteristics: (**a**) identification of actuation current, (**b**) correlation between current; force–deformation.

SMA actuators can generate relatively consistent responses when used in controlled circumstances; the repeatability is high, standard deviation is too low (virtually invisible) [28]. A current transducer, a miniature load cell, and a laser displacement sensor are configured properly to measure the activation current given to the actuator, the physical variables of the actuator, the force, and the displacement. Tests are carried out multiple times to find the force and deformation of the SMA wire actuator; the averages of the results are considered for the analysis. The force exerted by the SMA is determined for various operating currents as in Figure 6a and the force-deformation characteristics of the SMA wire for the excitation current up to 0.3 A are shown in Figure 6b. It has been observed that hysteresis is minimal at lower magnitudes of current. The activation current to provide low hysteresis is thus established to be 0.3 A, and it is determined that the maximum force displayed by the SMA at this nominal value of current is 2.8 N. As the current increases force exerted via the SMA wire increases, the length of the wire decreases, causing forced displacement of the drive link of the compliant mechanism.

### 4.2. Characteristic Features of the Compliant Mechanism

The compliant mechanism that consists of elastic links with a drive link is connected to the gripping arms symmetrically; the top symmetrical axis is depicted in Figure 2b. Here, $l_{g}\;$ is the distance from the axis of rotation to the point of application of force anddistance $a_{h}\;$ is given in

(12) $$a_{h}=l_{g}sin\theta_{g}$$

Torque is then obtained as follows:

(13) $$Torque=F_{in}\cdot a_{h}=F_{in}\cdot l_{g}sin\theta_{g}$$

**Figure 7:**
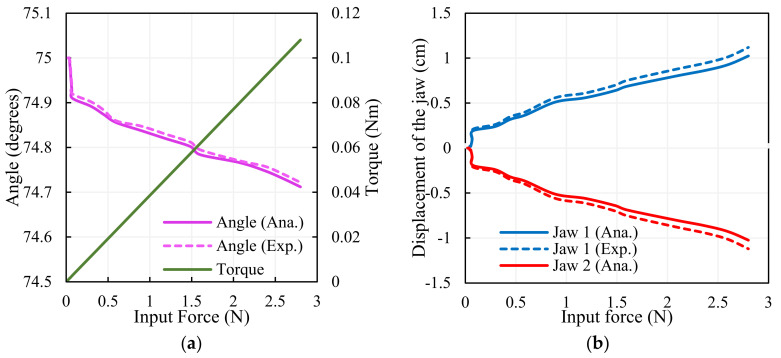
Characteristics of the gripper: (**a**) angle–torque of the gripper. (**b**) Displacement of the gripper jaws.

The results of the output angle and torque for the input force are depicted in Figure 7a; the analytical values match the experimental results. The gripper arms move with the same magnitude in a different direction due to the force exhibited by the SMA wire; the analytical and experimental findings of the displacement of the jaws are illustrated in Figure 7b; the results are in coincidence with each other.

## 5. Principle and Operation of the SMA Gripper

For the object to be grasped, any simple gripping devi ce must always have at least two fingers. Various works of the literature concerned with SMA-actuated grippers reveal that activating SMA causes the jaws to close (grasping mode) and the biasing element that acts in opposition causes the jaws of the gripper to open (release mode). In this contribution, when the SMA wire is energized, the SMA is in actuation mode; the arms of the gripper come closer and the object is held between the arms creating the grasping mode. In the release mode of operation, when the SMA is deactivated, the elastic arm of the gripper provides bias to the SMA wire, avoiding an additional biasing unit for the gripper. This self-biased structural design makes the gripping assembly compact and simple. Both the jaws of the developed gripper are movable and are connected to a common T-link to ascertain the equal distribution of force to both the arms of the gripper. This configuration simplifies the design and improves the grasping accuracy. The working principle of the gripper lies in transforming the linear displacement of the SMA wire actuator into an angular displacement at the jaws of the gripper. When the SMA wires are actuated using joule heating, the force exerted by the wire contracts and pulls the pseudo-rigid body that causes angular displacement in both the upper and lower jaws of the gripper. The upper and lower jaw displaces in opposite directions to close the gripper, creating a grasping mode that enables the holding of objects. During deactivation, the SMA wire stretches with the assistance of the elastic recovery force displayed by the compliant mechanism, which helps the gripping jaws release the object. The SMA wire is activated with a linearized current of 0.3 A that exhibits a maximum pull force of 2.8 N by deforming 3% of its length. The SMA-integrated compliant mechanism is a monolithic soft gripper that transmits motion with displacement amplification.

A direct means to measure the performance characteristics of the mechanism is to use the theories of geometric advantage and mechanical advantage, as specified in [29]. The fabricated gripper is analyzed for its performance by investigating the mechanical advantage (*MA*) and geometric advantage (*GA*). The output force *F_out_* is measured using the Force-Sensitive Resistor (FSR) and the input–output displacements (*U_in_*, *U_out_*) are measured using a laser displacement sensor for the computations of the performance of the gripper. Assuming that there is no friction in the system, the mechanical advantage of a system is the ratio of the force that achieves the useful work to the force applied. The extent to which the mechanical advantage differs from the theoretical value in actuality depends on the amount of friction. Mechanical advantage and geometrical advantage of the gripping assembly are expressed in Equations (14)–(17). The mechanical advantage of mechanical system is given using

(14) $$MA=F_{Out}/F_{in}$$

The geometrical advantage of the mechanical system is the ratio of the output displacement (*U_out_*) that performs the useful work to the input displacement (*U_in_*).

(15) $$GA=U_{Out}/U_{in}$$

**Figure 8:**
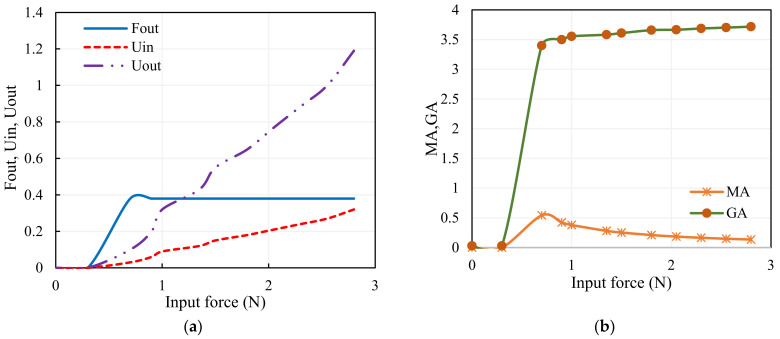
Mechanical features of the gripper: (**a**) characteristics of the SMA-actuated compliant gripper, (**b**) performance of the gripper.

The characteristics of the SMA wire-actuated, monolithic compliant gripper-like input displacement due to the force exhibited by the SMA wire, the output force at the gripper arms, and the output displacement are portrayed in Figure 8a, and its performance is depicted in Figure 8b. The monolithic compliant gripper offers the constant force output, and it is seen that the *GA* increases as the input force increases at the expense of *MA*, which leads to greater mechanical efficiency.

## 6. Controlled Implementation of the Gripping System

### 6.1. Self-Sensing Actuation

Grippers are the end-effectors that act as a final tool and have mechanical contact between robots and the entity to be grasped. These are the key component of robots and are essential to numerous manipulation operations. Grippers make close contact with the objects they are manipulating. Safe grabbing necessitates harmless physical contact with the object being grabbed, and the gripper must possess the ability to avoid any slippage. To enable secure grips for objects in a variety of forms, sizes, and materials, appropriate control algorithms are required. Closed-loop micromanipulation requires various sensors to detect process parameters to execute the control task accurately and quickly. Unfortunately, due to their sizes, performances, and restricted ability to detect the changes, these sensors are not well suited for the miniaturized era. As a result, a different approach is to use the actuators in sensing mode by recording changes in electrical resistance during their actuation using the self-sensing concept. The self-sensing approach has several benefits over the usage of external sensors. The concept enables a reduction in cost by getting rid of pricy sensors and the resolution and sensitivity can be equivalent to that of other sensors. Self-sensing is based on energy extraction in the form of an electrical variable; in reality, displacement is almost proportional to the output voltage, i.e., from a mechanically moving component, where the requisite exact variation in the electrical component of an actuating element is created. The idea of merging sensing and actuating capabilities (sensaction) in a single device gives rise to self-sensing technology. This technique incorporated in this article is to determine the displacement of the gripper from the change in the electrical resistance of the SMA wire. 

**Figure 9:**
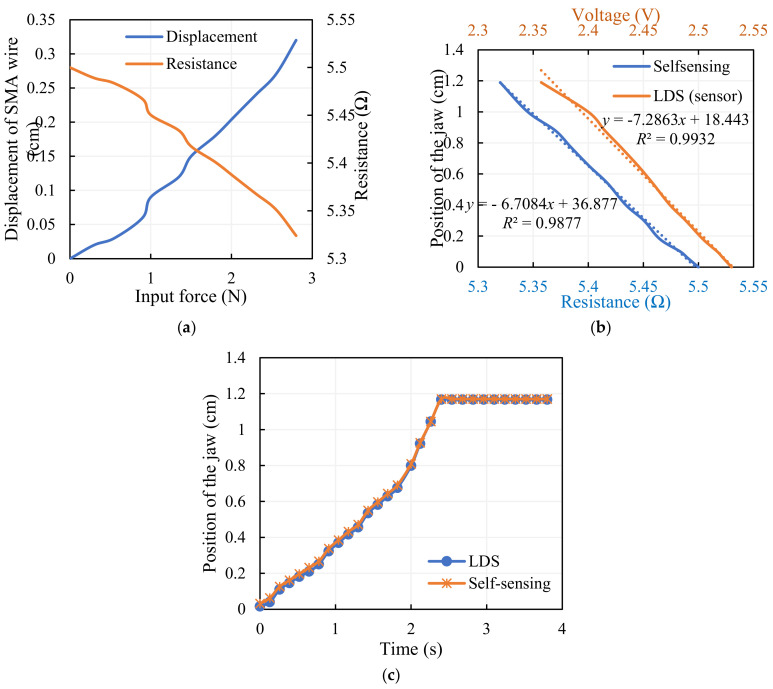
SMA wire as a self-sensor: (**a**) change in the characteristics of SMA wire with varying force, (**b**) performance of sensor (LDS) and self-sensor, (**c**) validation of self-sensing with LDS.

The deformation of the SMA wire and its corresponding electrical resistance concerned with the force exhibited by the SMA wire is seen in Figure 9a. Electrical resistance is evaluated by monitoring the voltage and current flowing through the wire for the activation current and is directly correlated to the displacement at the gripper arm. This self-sensing phenomena of the SMA wire provides the estimate of displacement in terms of electrical resistance, as depicted in Figure 9b and given using Equation (16), and the quantity is validated with a standard laser displacement sensor, as in Figure 9c. This sensor-less method of calculating the gripper’s displacement opens up the potential of building a miniature measurement device. A complete controlled gripping system is developed by the implementation of an appropriate control technique.

(16) $$y=-6.7084x+36.877\;,R^{2}=0.9877\;$$

A standard laser displacement sensor (LDS) is used to validate the self-sensing system; the voltage variation of the standard LDS for the displacement of the gripper arm is shown and the displacement of the gripper is estimated from the voltage output, as given in Equation (17).

(17) $$y=-7.2863x+18.443,\;\;\;\;\;\;\;R^{2}=0.9932$$

Equations (16) and (17) from the trendline of the plots represent the linear relationship between the measured resistance using the self-sensing approach and the position of the jaw, and the measured voltage using the laser displacement sensor and the position of the jaw, respectively. A fairly strong correlation coefficient (*R*^2^) of more than 0.98 validates the self-sensing procedure. It is seen from Figure 9c that the self-sensing data is in close agreement with the standard sensor, and the position of the gripper converges at 1.17 cm after a period of time due to the maximum input displacement (0.32 cm) and the contact between the jaws.

### 6.2. System Identification and Implementation of Control Strategy

**Figure 10:**
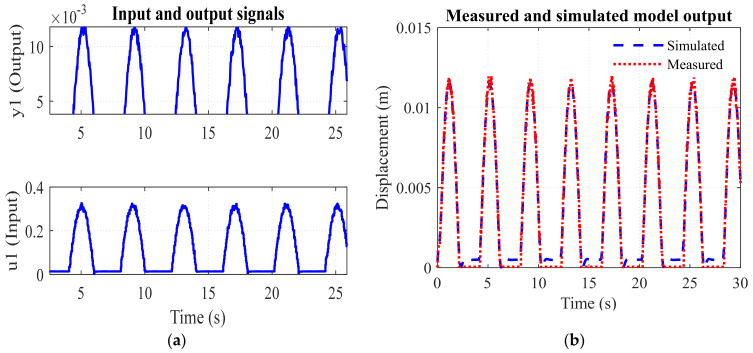
Signals associated with system identification: (**a**) input–output signals, (**b**) measured and simulated output.

Available, constitutive and thermo-mechanical models that characterize the actuation of the SMA wire actuator are difficult to implement for complex nonlinear systems. So, the SMA actuator system model is established from the experimental data collected by operating the gripper for various cycles. The input to the gripping system is the current through the SMA and the output is the displacement of the arm. In other words, the system uses the simplest cascade configuration in which the output force of the SMA is fed to the drive link and then transferred as displacement to the gripping arm. Hence the transfer function is given using Equation (18).

(18) $$\frac{Y_{1}\left(s\right)}{I\left(s\right)}*\frac{Y\left(s\right)}{Y_{1}\left(s\right)}=\frac{Y\left(s\right)}{I\left(s\right)}$$

where *I*(*s*), $Y_{1}\left(s\right)$, and Y(s)  are the Laplace transforms of the activation current, displacement of the drive link (SMA), and displacement of the gripping arm, respectively. The system identification toolbox in MATLAB^®^ Simulink is used for the identification of the gripping system model. Input–output signals of the gripper are measured experimentally as shown in Figure 10a and fed as time-domain signals to identify the system. Half of the data is taken into account for identification, while the other half is taken into account for validation; the measured and simulated outputs are depicted in Figure 10b. The second order transfer function model of the SMA-actuated gripper is obtained, as in Equation (19).

(19) $$\frac{Y\left(s\right)}{I\left(s\right)}=\frac{2.59}{s^{2}+9.846\;s+71.27}$$

Transfer function model is identified with fit to estimation data = 91.42% and the final prediction error = 0.0000382.

**Figure 11:**
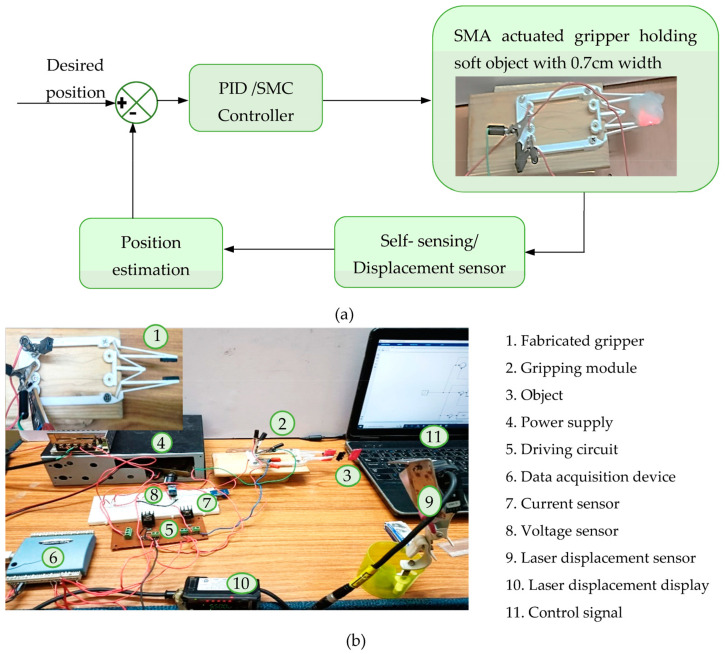
Implementation of control strategy: (**a**) schematic, (**b**) experimental setup.

Researchers have considered linear controllers as well as approaches like optimum control, fuzzy logic, and pulse width modulation in the controls field relating to shape memory alloy actuators as stated by [30]. The SMA actuators configured to perform finger action can be bent or stretched independently by actuating the corresponding actuator, and the motion can be controlled by applying coordinated control of the SMA actuators using the proportional–derivative (PD) controller [31]. Variable structure/sliding mode control is investigated by various researchers including [32,33], and some encouraging outcomes have been revealed among them. The schematic and the experimental setup for the implementation of the control strategy are depicted in Figure 11a,b.

A proportional–integral–derivative (PID) control scheme is implemented for the control of the position of the jaws of the gripper for the comparative analysis of the performance of the controllers. The control signal of the dominant, well-known PID controller is given in Equation (20).

(20) $$u\left(t\right)=K_{p}e\left(t\right)+K_{i}\int e\left(t\right)dt+K_{d}\frac{\;d}{dt}e\left(t\right)$$

where, $K_{p}$, $\;K_{d}$, and $\;K_{i}$ are proportional, derivative, and integral gains, respectively, and *e*(*t*) is the error signal. A sliding mode control law offered for the gripper mechanism uses the linear first-order sliding surface and is based on the system model. In this section, a sliding mode controller is designed to maintain the desired movement by applying the required current to the SMA wire. The system is compared with the conventional PID controller for its performance. The states of the system are given using

(21) $$x_{1}=y\left(t\right)=y$$

(22) $${\dot{x}}_{1}=x_{2}=\dot{y}\;\left(t\right),\;\;{\dot{x}}_{2}=x_{3}=\ddot{y}\;\left(t\right)$$

Rearranging the transfer function of the system, the following equation is obtained:

(23) $$2.59\;R\left(s\right)=Y\left(s\right)\left(s^{2}+9.846\;s+71.27\right)$$

Then, after taking the inverse Laplace transform, the state-space representation of the system can be represented as follows:

(24) $$\left[\begin{array}{c} {\dot{x}}_{1}\\ {\dot{x}}_{2} \end{array}\right]=\left[\begin{array}{cc} 0 & 1\\ -71.27 & -9.846 \end{array}\right]\left[\begin{array}{c} x_{1}\\ x_{2} \end{array}\right]+\left[\begin{array}{c} 0\\ 2.59 \end{array}\right]u;\;\;y=\left[\begin{array}{cc} 1 & 0 \end{array}\right]\left[\begin{array}{c} x_{1}\\ x_{2} \end{array}\right]$$

Now, the above state-space can be generalized using

(25) $$\;\left[\begin{array}{c} {\dot{x}}_{1}\\ {\dot{x}}_{2} \end{array}\right]=\left[\begin{array}{cc} 0 & 1\\ -a_{1} & -a_{2} \end{array}\right]\left[\begin{array}{c} x_{1}\\ x_{2} \end{array}\right]+\left[\begin{array}{c} 0\\ b \end{array}\right]u;\;\;y=\left[\begin{array}{cc} 1 & 0 \end{array}\right]\left[\begin{array}{c} x_{1}\\ x_{2} \end{array}\right]$$

where $\;a_{1}$ = 71.27; $a_{2}$ = 9.846;  b = 2.59. The sliding surface *σ* (*t*) is chosen as the first-order differential equation of the error signal, and the solution is *e*(*t*) = 0, as stated in [34]:

(26) $$\sigma \left(t\right)=\left[{\left(\frac{d}{dt}+\lambda \right)}^{n-1}e\left(t\right)\right]$$

(27) $$\sigma \left(t\right)=\left[\dot{e}\left(t\right)+\lambda e\left(t\right)\right]$$

where *n* is the order of the system, and *e*(*t*) = $\;ref.-y=ref.-x_{1}.\;$ The variable *ref*. is the reference input. To maintain the sliding motion on the surface, σ˙(*t*) = 0, and substituting the expressions from the state-space model of the system, the equivalent sliding mode controller is obtained as follows:

(28) $$\;u_{s}\left(t\right)=a_{1}x_{1}\left(t\right)+\left(a_{2}-\lambda \right)x_{2}\left(t\right)$$

Now, in order to satisfy the sliding mode condition, the discontinuous part given in Equation (29) is added to Equation (28) to design a total sliding mode controller (SMC), which is robust against uncertainties and disturbances.

(29) $$\;\;u_{r}\left(t\right)=\beta sat\left(\sigma \left(t\right)\right)$$

This abrupt addition of the signal may cause the system to reach the reaching phase. The tuning parameters of the controllers are given in Table 2.

**Figure 12:**
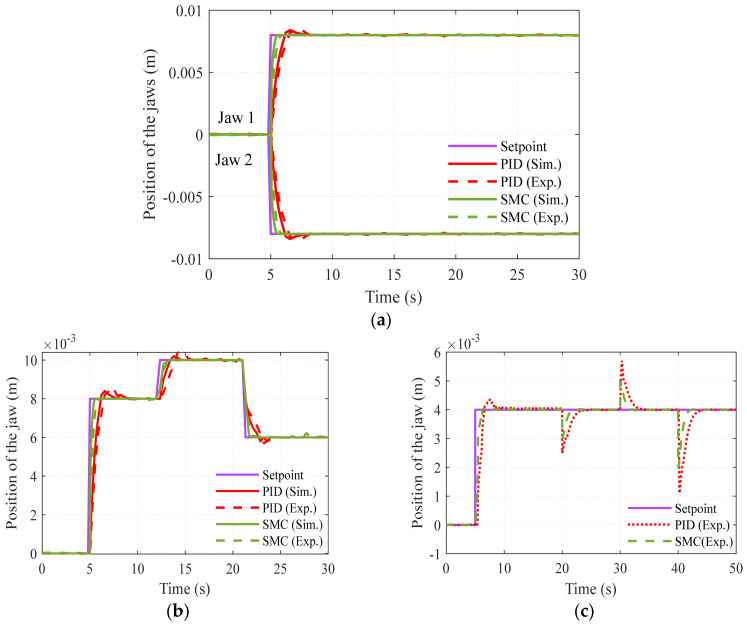
Controlled response: (**a**) step response, (**b**) multi step response, (**c**) disturbance rejection.

The controlled response of the system for step input, to set the jaws of the gripper at 0.008 m, is portrayed in Figure 12a; the jaws of the gripper move in opposite directions, while the excitation current to the SMA wire is of the same magnitude and polarity. The response for multistep input to set the gripper jaws at 0.008 m at 5 s, 0.01 m at 12 s, and 0.006 m at 22 s are acquired with the PID and SMC controllers, and is depicted in Figure 12b. The disturbance rejection ability of the controller is also checked with the designed controllers on the gripping system by setting the desired position at 0.004 m and providing disturbance (by pulling the jaw in both directions) at 20 s, 30 s, and 40 s, and it is demonstrated that the SMC controller performs better in terms of the rise time and settling time, etc., and the result is depicted in Figure 12c. The performance of the system with various controllers is tabulated in Table 3 and it is determined that the SMC performs better than classical PID in terms of peak overshoot, steady state error, and response time. It is also inferred that the disturbance rejection capability is more for the SMC than the PID, and that SMC is best suited for SMA-actuated systems.

## 7. Conclusions

In this work, a small-sized gripper using the SMA wire incorporated with the compliant mechanism is developed, and its performances such as the deformation of the SMA wire and position controllability of the jaws are evaluated through both simulation and experiment. The feasibility of designing a single piece monolithic gripping structure with a defined form of a compliant mechanism associated with SMA wires is investigated in this paper. The functionality of the gripper is exemplary due to the unique capability of the smart material SMA. The possibility of designing a monolithic structure to change its position in a defined manner based on the sensacting functionality of the SMA was explored with an appropriate control strategy. The implemented gripper herein permitted repetitive manipulations and could provide an effective means of handling micro/macro scale robotic systems. The gripping force and the capture range is experimentally tested, and the results show the constant output force of 0.38 N, the jaw opening up to 0.012 cm, and its corresponding current values. The compliant gripping arms provide the bias force necessary for the cyclic operation of the SMA gripper. 

Some major results having scientific significance in this research field are summarized as follows. The SMA wire actuator is configured as a loop to offer more strain and compactness of the gripper; the dimensions of the SMA wire, the length and the diameter, are appropriately selected as 10 cm and 0.15 mm to deliver the requisite stroke of 0.32 cm and a pulling force of 2.8 N. The activation current to provide the necessary force with linear phase transformation is investigated through an experimental study, and it is found that the transformation cycle for the current of 0.3 A presents linear behaviour while exhibiting the necessary force. The controlled action of the SMA-based gripper is verified through the sliding mode control strategy, and its performance and disturbance-rejection capability is better than the PID control. It is finally remarked that various gripping claw designs would be explored in future for holding a wider range of targets with an optimal gripping control strategy. 

This gripping mechanism uses a monolithic construction that avoids movable parts, and hence there are no friction problems. The ABS material used for the fabrication of the gripper withstands the high temperature of the SMA wire actuator and the design enables self-biased operation. Displacement initiated at the input end by the SMA wire is delivered with a displacement gain of 3.7 at the output end, due to the displacement amplification of the compliant mechanism; this improves the capture reliability and the geometric advantage of the gripping module. The gripper design with dimensions 0.09 m × 0.04 m captures objects with a maximum size of 0.012 m weighing up to 35 g. However, the limitations of using the SMA wire actuator are the high current and limited bandwidth due to the long cooling time.

## Figures and Tables

**Table 1:** Dimensions and specifications of the constituents of the monolithic compliant gripper.

SMA Wire Actuator		Gripping Arm	
Material	NiTiNOL	Material	ABS
Diameter (m)	10.15 × 10^−3^	Length (m)	0.04
Length (m)	0.10	Width (m)	0.004
Pull force (N)	3.14	Thickness (m)	0.001
Density (kg/m^3^)	6450	Density (kg/m^3^)	1000
Resistance (Ω/m)	55	Stiffness (N/m)	31.25
Transition temperature (°C)	70	Young’s modulus (GPa)	2

**Table 2:** Tuning parameters of the controllers.

Controller	*K_p_*	*K_i_*	*K_d_*	λ	β
**PID**	1.1	1.4	0.2	-	-
**SMC**	-	-	-	0.634	500

**Table 3:** Performance of the controllers.

Performance	PID	SMC
Rise time (s)	1.5	0.61
Settling time (s)	2.4	0.78
Peak overshoot (%)	3.75	0
Steady state error	0.023	0

## Data Availability

All data achieved in this work are available by requesting from the potential readers.
